# From Airways to Arteries: Dissecting the Inflammatory Mechanisms of Pulmonary Vascular Remodeling in a Murine Model of Chronic Airway Inflammation

**DOI:** 10.3390/biomedicines14061359

**Published:** 2026-06-17

**Authors:** Silvia Siragusa, Elena Tantillo, Silvia Parolo, Gloria Modafferi, Maria Laura Faietti, Giulia Natali, Paola Caruso, Sofia Beghi, Silvia Cantoni, Mary Delli Carpini, Maria Giulia Gualandri, Antonella Maria Nogara, Costanza Anna Maria Lagrasta, Vanessa Pitozzi, Maurizio Civelli, Gino Villetti, Enrico Domenici, Marcello Trevisani, Barbara Pioselli, Silvia Pontis

**Affiliations:** 1 Department of Medicine and Surgery, University of Parma, 43126 Parma, Italy; s.siragusa@chiesi.com (S.S.); antonellamaria.nogara@unipr.it (A.M.N.); costanzaannamaria.lagrasta@unipr.it (C.A.M.L.); 2 Global Research and Preclinical Development, Chiesi Farmaceutici S.p.A, 43122 Parma, Italy; e.tantillo@chiesi.com (E.T.); s.parolo@chiesi.com (S.P.); g.modafferi@chiesi.com (G.M.); ml.faietti@chiesi.com (M.L.F.); g.natali@chiesi.com (G.N.); plcaruso63@gmail.com (P.C.); s.beghi@chiesi.com (S.B.); s.cantoni@chiesi.com (S.C.); dellicarpini.mary@gmail.com (M.D.C.); mg.gualandri@chiesi.com (M.G.G.); v.pitozzi@chiesi.com (V.P.); m.civelli@chiesi.com (M.C.); g.villetti@chiesi.com (G.V.); m.trevisani@chiesi.com (M.T.); 3 Fondazione the Microsoft Research-University of Trento Centre for Computational and Systems Biology (COSBI), 38068 Rovereto, Italy; domenici@cosbi.eu; 4 Department of Chemistry, Life Sciences and Environmental Sustainability, University of Parma, 43124 Parma, Italy

**Keywords:** in vivo models, COPD, PH, chronic inflammation, hypoxia, hypersecretion of mucus, small pulmonary vascular remodeling

## Abstract

**Background**: Chronic Obstructive Pulmonary Disease (COPD) is a progressive, incurable condition marked by irreversible airflow limitation and systemic inflammation. Cardiovascular comorbidities, particularly pulmonary hypertension (PH), exacerbate disease severity. While cigarette smoke is a well-known trigger, non-smoking-related inflammatory pathways remain underexplored. This study investigates vascular remodeling in a murine model of inflammation induced by chronic exposure to house dust mite Farinae (HDM). **Methods**: Female C57BL/6 mice were sensitized with HDM in Freund’s Complete Adjuvant and challenged intranasally with HDM for six weeks. Lung inflammation, mucus hypersecretion, and vascular remodeling were evaluated via BAL, histology, immunofluorescence, echocardiography, gene expression, proteomics, and FlexiVent pulmonary function tests (FlexiVent system). **Results**: HDM exposure induced a mixed inflammatory response, with elevated neutrophils, monocytes, and lymphocytes in BALF. Mucus hyperproduction (increase in MUC5AC/MUC5B) and impaired lung function (reduced FEV0.1/FVC) were observed. Vascular remodeling was evidenced by increased wall thickness, α-SMA expression, and collagen deposition. Proteomic analysis revealed dysregulation of endothelial markers and protease/antiprotease imbalance. HIF1-α was significantly upregulated in lung tissue and correlated with vascular and epithelial remodeling. **Conclusions**: Chronic HDM exposure in mice recapitulates key features observed in subsets of COPD and PH, including inflammation-driven airway and vascular remodeling. HIF1-α emerges as a central regulator, linking hypoxia to structural changes. This model offers insights into the effect of non-smoking-related inflammatory pathways on bronchial and vascular remodeling that are potentially relevant for subgroups of COPD patients and highlights HIF1-α as a potential therapeutic target.

## 1. Introduction

Chronic obstructive pulmonary disease (COPD) is a progressive disorder characterized by irreversible airflow limitation. According to the World Health Organization, it ranked as the fourth leading cause of death globally in 2021, responsible for approximately 3.5 million deaths, around 5% of all fatalities worldwide. COPD is a complex disease in which chronic inflammation, mucus overproduction, small-airway remodeling and emphysematous parenchyma destruction coexist [1]. Despite a similar degree of airflow obstruction, substantial heterogeneity in the clinical manifestations and outcomes are observed. Comorbidities like cardiovascular diseases (CVDs), osteoporosis, muscle weakness, and lung cancer accelerate COPD’s progression and introduce additional elements of clinical diversity [2]. CVD increases the risk of both exacerbations and mortality in patients with COPD and, among patients with moderate COPD, deaths due to cardiac conditions exceed those caused by respiratory failure [3]. Patients diagnosed with COPD and CVD experience worse quality of life with higher rates of hospitalization, representing an economic burden for the country [4,5,6,7]. Such evidence suggests that close attention should be paid to the relationship between pulmonary and cardiovascular events in this pathology [8,9,10]. Among CVD, pulmonary hypertension (PH) is one of the most common comorbidities affecting COPD patients, with an estimated overall prevalence of 39% according to the systematic meta-analysis performed by Zhang and colleagues [11] COPD-PH lacks specific therapeutic options due to incomplete understanding of its pathogenesis. PH was suggested to be the result of hypoxia associated with emphysema and vascular pruning; however, structural abnormalities of pulmonary arteries are not exclusive of severe COPD, as they have also been found in patients with mild COPD and even in smokers with normal lung function [12,13]. In rodent models of cigarette smoke exposure, pulmonary vascular changes with neo-muscularization of precapillary arteries preceded the development of emphysema [14,15], suggesting that cigarette smoke per se can lead to vascular remodeling [16]. Chemicals in cigarette smoke directly injure lung endothelial cells, causing endothelial dysfunction (loss of nitric oxide, excess endothelin-1), cell death and barrier disruption [17]. These changes trigger vasoconstriction and abnormal growth of vessel wall cells, leading to structural alterations. Although the pathophysiology of smoke-induced vascular remodeling has been widely characterized, the mechanisms driving vascular remodeling in non-smoking COPD-PH patients have been poorly explored. Therefore, in this study, we investigated the effect of long-term mixed pulmonary inflammation on vascular and bronchial epithelial remodeling. The sensitization of mice with house dust mites (HDMs) in complete Freund’s adjuvant (CFA), followed by repeated HDM challenges over a 6-week period, reproduced key features observed in a subset of COPD patients with a mixed inflammatory profile, mucus hyperproduction and impaired lung function. Moreover, the allergenic stimulation caused vascular alterations. This model facilitates the investigation of inflammatory signaling pathways that contribute to vascular and epithelial remodeling in the context of COPD-associated pulmonary hypertension.

## 2. Materials and Methods

### 2.1. Animals

Female C57BL/6 mice (weighing 20–25 g, 8–10 weeks of age) were purchased from Envigo (Udine, Italy) and housed in standard light conditions (12 h light/dark cycle) with food and water ad libitum. Animals were monitored twice daily to assess general health status and clinical signs, while body weight was recorded every two days. Humane endpoints were defined prior to study initiation. In accordance with the protocol approved by the Ministry of Health, animals exhibiting a body weight loss greater than 20% or displaying signs of severe clinical deterioration were removed from the study to prevent discomfort. All the experimental procedures involving mice were conducted in an AAALAC (Association for Assessment and Accreditation for Laboratory Animal Care)-certified facility and were approved by the local ethics committees, authorized by the Italian Ministry of Health in full compliance with the international European ethics standards of directive 2010/63/EU, Italian D. Lgs. 43 26/2014, the revised “Guide for the Care and Use of Laboratory Animals” (Guide for the Care and Use of Laboratory Animals and National Research Council, 2010) and the ARRIVE guidelines (Animal Research: Reporting of In Vivo Experiments).

### 2.2. Chemicals

Sensitization was induced by the subcutaneous (sc) administration of 100 µL of an emulsion containing 50 µL of house dust mite Farinae extract (HDM) (3.3 mg/mL, Stallergenes Greer, Lenoir, NC, USA) in saline solution 0.9% (Sodium Chloride 0.9%, Eurospital S.p.A, Trieste, Italy) and 50 µL of Freund’s Complete Adjuvant (CFA) (Sigma-Aldrich, St. Louis, MO, USA) to each mouse. Allergen challenge was performed by intranasal instillation (IN) of 30 µL of HDMs in saline solution (0.8 mg/mL) for each animal. Mice were anesthetized with 4% Isoflurane (Zoetis, Kalamazoo, MI, USA) before HDM administration. Additionally, Pentothal sodium solution (1 g/50 mL) (MSD Animal Health, Rahway, NJ, USA) was administered as anesthesia before euthanasia. To perform lung function analysis with the Flexivent instrument, Ketamine (100 mg/kg, Lobotor, ACME Srl, Cavriago, Italy), Xylazine (10 mg/kg, Rompun, Bayer S.p.A, Leverkusen, Germany) and Pancuronium (1 mg/kg, Sigma, St. Louis, MO, USA) were used to facilitate tracheal intubation and to prevent spontaneous breathing. To execute broncho-alveolar lavage (BAL), BAL fluid (BALF) solution consisting of 10X HBSS (Hanks’ Balanced Salt Solution, Thermo Fisher Scientific, Waltham, MA, USA), 10 mM Ethylenediaminetetraacetic acid (EDTA, Fluka analytical, Sigma-Aldrich, St. Louis, MO, USA), 10 mM 4-(2-hydroxyethyl)-1-piperazineethanesulfonic acid (HEPES, Gibco, ThermoFisher Scientific, Waltham, MA, USA) and distilled water (Sigma-Aldrich, St. Louis, MO, USA) was used. Explanted cardiac tissues were submerged in a Krebs solution using KCl (Sigma-Aldrich, St. Louis, MO, USA) 8 g/L, MgSO_4_ × 7H_2_O (Sigma-Aldrich, St. Louis, MO, USA) 0.29 g/L, KH_2_PO_4_ (Sigma-Aldrich, St. Louis, MO, USA) 0.16 g/L, CaCl_2_ × 2H_2_O (Sigma-Aldrich, St. Louis, MO, USA) 0.18 g/L, NaHCO_3_ (Sigma-Aldrich, St. Louis, MO, USA) 2.1 g/L and Glucosio × H_2_O (Sigma-Aldrich, St. Louis, MO, USA) 2 g/L.

### 2.3. Experimental Protocol

After the acclimatization period, mice (total *n* = 54) were stratified by body weight and subsequently randomized into two experimental groups using a computer-generated allocation scheme, ensuring comparable baseline body weight distributions between groups. Cage positions were periodically rotated within the animal facility to minimize potential location-related biases and ensure equal exposure to environmental conditions.

**Figure 1:**
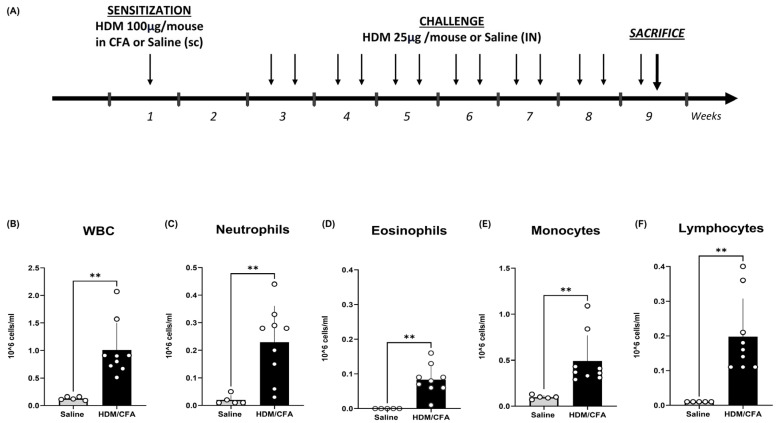
Experimental protocol and inflammatory cell response in BALF of HDM/CFA-stimulated mice. (**A**) Experimental protocol. (**B**–**F**) Total inflammatory cells (WBCs), neutrophils, eosinophils, monocytes and lymphocytes in BALF of mice treated with HDM/CFA or saline solution. Data are shown as mean ± SD. Statistical analysis was carried out using unpaired Student’s *t*-test in comparison with saline group. ** *p* < 0.01.

The first experimental group, hereafter called HDM/CFA (total number of animals used *n* = 29), was sensitized with a subcutaneous (sc) injection of an emulsion formed by HDM in CFA on day 0 (week 1), and subsequently, from week 3 to week 8, mice received a challenge by intranasal (IN) instillation of 30 µL of HDMs in saline solution twice a week, according to the experimental protocol (Figure 1A). The second group received saline solution in both sc and IN administrations as control (total number of animals used *n* = 25). The experimental protocol was conducted over 8 weeks, as previously described by Ouyang and colleagues [18]. Twenty-four hours after the last challenge administration, mice were deeply anesthetized using Pentothal sodium solution and euthanized via abdominal aorta bleeding. For each experiment, a maximum of *n* = 10 animals for the saline group and *n* = 15 for the HDM/CFA group were used in accordance with the ministerial authorization n. 19/2025-PR. The sample size was determined using G*Power software (Version 3.1.9.2; Franz Faul, University of Kiel, Kiel, Germany) and GraphPad Prism (version 10.6.1; GraphPad Software, San Diego, CA, USA). Calculations were based on a one-way ANOVA with a power of 0.8, an effect size (f) of 0.8, and an α level of 0.05 across equally sized groups. We reduced the number of animals needed while allowing for proper statistical comparison to adhere to the 3Rs principles of Reduction and Refinement.

### 2.4. Broncho-Alveolar Lavage (BAL)

BALF was gathered to assess the inflammatory response; mice (saline *n* = 5 and HDM/CFA *n* = 9) underwent BAL by gently washing the lungs three times with 0.6 mL of clear sterile BALF passed through a catheter inserted into the trachea. An incision on the front of the neck was made to ensure direct access to the trachea [19]. The recovered volume of BALF was centrifuged at 400 rpm for 10 min at 4 °C. The supernatant was collected and cell pellets were resuspended in 200 µL of BALF. Total White Blood Cells (WBCs) and the differential cell counts of inflammatory cells (neutrophils, eosinophils, monocytes, and lymphocytes) were counted using a hemocytometer XN-1000 Automated Haematology Analyzer (Sysmex Corporation, Hamburg, Germany) and expressed in 10^6^ cells/mL.

### 2.5. Cytokine Analysis

After conducting the BAL procedure, lungs were removed, flash-frozen in liquid nitrogen and homogenized in PBS 1X (ThermoFisher Scientific, Waltham, MA, USA) added with a protease and phosphatase inhibitor cocktail (Sigma-Aldrich, St. Louis, MO, USA). For protein extraction, Cell Lysis Buffer (R&D, Minneapolis, MN, USA) was added according to the protocol. The lung protein lysate was then used to analyze cytokines of interest using the Luminex BioPlex-200 analyzer (MILLIPLEX MAP mouse Cytokine/Chemokine magnetic kit, Millipore, Burlington, MA, USA). The results are expressed as pg/mL.

### 2.6. Lung Processing for Gene Expression and Proteomic Analysis

A dedicated group of mice (saline *n* = 5 and HDM/CFA *n* = 5) was used to perform both gene expression and proteomic analysis. Transcardiac perfusion with saline solution (0.9% NaCl) was necessary to eliminate blood that could interfere with the method of analysis. Following perfusion, lungs were explanted and lobes separated; the right ones were flash-frozen in liquid nitrogen and stored at −80 °C for proteomic analysis. Left lobes were immediately collected in RNALater^®^ (Millipore, Burlington, MA, USA) according to the manufacturer’s instructions and stored for 5 days at 4 °C before RNA extraction.

#### 2.6.1. RNA Extraction and Gene Expression Analysis

Left lobes (saline *n* = 3 and HDM/CFA *n* = 3) were homogenized in QIAzol^®^ Lysis Reagent (Qiagen, Hilden, Germany) according to the manufacturer’s protocol using the TissueLyser II instrument (Qiagen, Hilden, Germany). RNA extraction was performed using the QIAcube automated system (Qiagen, Hilden, Germany) with the miRNeasy Mini Kit (Qiagen, Hilden, Germany). RNA purity and concentration were established using Nanodrop 2000 (ThermoFisher Scientific, Waltham, MA, USA). For gene expression analysis, Syber Green technology was used to perform quantitative Real-Time PCR (qPCR). For Syber Green-based qPCR, the First Strand Kit (Qiagen, Hilden, Germany) was used to obtain cDNA. The expression levels of a panel of genes spotted in custom RT2 PCR Arrays were evaluated using RT2 SYBR Green ROX qPCR Mastermix (Qiagen, Hilden, Germany). All qPCR analyses were performed using the Applied Biosystems QuantStudio 7 Flex PCR System (ThermoFisher Scientific, Waltham, MA, USA). Gene expression was determined using the 2^−ΔCt^ method, with normalization to the reference gene Heat shock protein 90 alpha family class B member 1 (HSP90Ab1). Fold changes were calculated relative to the saline group and the results presented as Log_2_ fold change.

#### 2.6.2. Proteomic Analysis

Right lobes of mice (saline *n* = 5 and HDM/CFA *n* = 5) preserved at −80 °C were homogenized in a mix of RIPA buffer (Sigma-Aldrich, St. Louis, MO, USA) and protease phosphatase inhibitor cocktail (Sigma-Aldrich, St. Louis, MO, USA) with TissueLyser II instrument (Qiagen, Venlo, The Netherlands). The extracted proteins were quantified with BCA assay. A total of 50 μg of each sample was digested for 16 h with Trypsin (1:20 *w*/*w*) following the S-trap micro protocol. Samples were analyzed with a Thermo Scientific Dionex Ultimate 3000 nano RSLC, Thermo Scientific, Germering, Germany, coupled with nanoESI source. A total of 1 μg of peptides (corresponding to a volume of 2 μL) was first trapped on a μ-precolumn 300 μm i.d. × 5 mm PepMap100, 5 μm, 100 Å, C18 (ThermoFisher Scientific, Waltham, MA, USA) for 3 min, at 10 μL/min and H_2_O 0.1% TFA. Peptides were then separated on a EasySpray (75 µm × 500 mm, 2 μm, 100 Å) (ThermoFisher Scientific, Waltham, MA, USA) heated to 40 °C at 300 nL/min using a gradient of 5–50% eluent B (acetonitrile, 0.1% formic acid) for 240 min and 50–85% eluent B for 5 min followed by 20 min at 85% eluent B for washing and 30 min equilibration. The mass spectrometer was operated in data-dependent acquisition (DDA) mode. The following MS2 analysis was conducted with a top-speed approach. The most abundant ions were selected for fragmentation by collision-induced dissociation (CID). Reporter ions were detected using Orbitrap with a resolution of 30,000. The mass spectrometer worked in positive polarity mode and singly charge state precursors were rejected for fragmentation. Database research was performed with Proteome Discoverer v2.4 software (ThermoFisher Scientific, Waltham, MA, USA) using Sequest HT search engine and Mus musculus database (2022) and contaminants. The search was run against targeted and decoy databases to determine the false discovery rate (FDR). Search parameters included trypsin, allowing for two missed cleavage sites, carbamidomethyl in cysteine as static modification, methionine oxidation, and acetylation in protein N-terminus as dynamic modifications. Peptides with a q-value lower than 0.1 and an FDR < 1% were considered positive identifications with a high confidence level. Differential protein abundance analysis was performed using Log2-transformed values and a two-tailed unpaired *t*-test assuming equal variance across the two groups, as implemented in Perseus. Multiple test corrections were performed using the Benjamini and Hochberg (BH) method. Gene Ontology Biological Process (GO-BP) gene set enrichment analysis (GSEA) was conducted using the fgsea R package (v. 1.32.2). The mouse GO-BP gene set was downloaded from The Molecular Signatures Database (MSigDB) on 7 February 2025. For GSEA ranking, protein *p*-values from the differential abundance analysis were transformed as −Log10(*p*-value), and the resulting values were multiplied by the sign of the corresponding Log2 fold change (HDM/CFA vs. saline). The lung single-cell RNA sequencing (scRNAseq) dataset from Adams and colleagues [20] was downloaded from NCBI GEO (accession code: GSE136831). Cell type expression specificity analysis was performed using CELLEX (https://github.com/perslab/CELLEX, accessed on 7 February 2025; v.1.2.2). Only cells from COPD samples were included in the analysis. For this analysis, subtypes of endothelial, monocyte, and dendritic cell types were grouped into one main cell type. Genes were identified as cell-specific markers if they displayed a CELLEX specificity score greater than 0.8. Mouse orthologs of human genes were retrieved from Ensembl BioMart annotations (Release 113), accessed through the Ensembl web service. The list of proteases, antiproteases, and protease subclasses was retrieved from PANTHER (https://pantherdb.org/; accessed on 6 March 2025). The list of *HIF1-α* target genes was retrieved from the CollecTRI database (https://github.com/saezlab/CollecTRI; accessed on 10 September 2025), accessed through the decoupleR get_collectri function. Only positive *HIF1-α*–target gene interactions were selected.

### 2.7. Pulmonary Function Assessment

As previously described by Murgo et al. [21], respiratory system mechanics and the Pressure Volume relationship were assessed in a dedicated group of mice (Saline *n* = 10 and HDM/CFA *n* = 9), utilizing the FlexiVent System (SCIREQ Inc., Montreal, QC, Canada). Mice were anesthetized with an intraperitoneal injection of ketamine (100 mg/kg) and xylazine (10 mg/kg), in 1:1 ratio and subjected to tracheostomy. To prevent spontaneous breathing, Pancuronium (1 mg/kg) was also intraperitoneally injected. Each mouse was intubated using an 18-gauge metal endotracheal cannula and connected to the instrument. Ventilation was set at a respiratory rate of 150 breaths/min and a tidal volume of 10 mL/kg, with a positive end-expiratory pressure of 3 cmH_2_O to maintain a mean lung volume that mimics spontaneous breathing. The single forced oscillation technique (FOT) measurements were fitted to a single-compartment model to determine respiratory system resistance (Rrs) and respiratory system compliance (Crs). The multi-frequency FOT measurements were fitted to a constant phase-model to obtain the Newtonian resistance (Rn) and tissue elastance (H). Pressure Volume (PV) loops were also generated to obtain the compliance (I) of the respiratory system, an estimate of inspiratory capacity (IC), the curvature of the upper portion of the deflation limb of the PV curve (K) and the area enclosed by the PV loop (Area). The negative pressure-driven forced expiratory (NPFE) maneuver was then performed by inflating the mouse lungs to a pressure of 30 cm H_2_O over 1 s and holding this pressure for 2 s before connecting the animal’s airways to the negative pressure reservoir (−50 cm H_2_O) for 2 s. The forced expired volume over 0.1 s (FEV0.1) and the forced vital capacity (FVC) were calculated directly from the flow volume loop generated during lung deflation. All maneuvers and perturbations were performed until three acceptable measurements (coefficient of determination ≥ 0.95) were achieved. An average of the three measurements was calculated and depicted per mouse. Data analysis was conducted using FlexiVent software version 8.1.

### 2.8. Histology and Immunofluorescence Staining

A group of mice was dedicated to histological analysis (saline *n* = 10 and HDM/CFA *n* = 15). Animals were deeply anesthetized and transcardiac perfusion with saline solution 0.9% (Sodium Chloride 0.9%, Eurospital S.p.A, Trieste, Italy) was performed. Lungs were insufflated through a tracheal cannula with 10% formalin buffer, explanted and stored in the same buffer for 24 h at room temperature. Tissues were paraffin-embedded and cut to obtain longitudinal sections of 5 µm thickness that were used for histological and immunofluorescent (IF) staining. Subsequent sections were stained with Masson’s Trichrome, Hematoxylin and Eosin (H&E) and Alcian blue/PAS (Periodic Acid-Schiff) techniques (activities performed by Histolab Verona, Verona, Italy). Additional tissue slices (from saline *n* = 5 and HDM/CFA *n* = 5) were incubated with mouse-anti α-SMA (1:200, 90′, 37 °C; A5228, (Sigma-Aldrich, St. Louis, MO, USA),) and goat-anti CD31 (1:100, o/n, 4 °C; AF3628, R&D Systems, Minneapolis, MN, USA) primary antibodies, respectively. After, slices were incubated with anti-mouse IgG Tetramethyl Rhodamine Isothiocyanate (TRITC) and anti-goat IgG Fluoresceine Isothiocyanate (FITC)-conjugated secondary antibodies (1:20, 60′, 37 °C; T2402 and F7367, respectively, Sigma-Aldrich, St. Louis, MO, USA). Coverslips were mounted with fluorescence mounting medium with 4′,6-Diamidino-2-phenylindole for nuclei detection (DAPI, Vector Laboratories, Burlingame, CA, USA, H-1200). Histological preparations were digitalized through the image analysis system NDP scan 3.3 using a NanoZoomer S-60 scanner (Hamamatsu Photonics K.K, Hamamatsu City, Japan) at 20× magnification.

#### 2.8.1. Percent (%) Wall Thickness and Integrated Optical Density (IOD) to Quantify Vascular Remodeling

Vessel wall thickness (%) was assessed on small pulmonary vessels with an external diameter between 20 and 150 μm on lung parenchyma sections stained with H&E. For each animal (saline *n* = 8 and HDM/CFA *n* = 8), 10–11 small vessels were counted within a single region of approximately 40–60 mm^2^. For each vessel, wall thickness and the external diameter were measured twice, along with two different axes. Percent wall thickness was calculated as [wall 1a thickness + wall 1b thickness)/external diameter 1] × 100. The calculation was repeated on both axes and the two values were averaged to obtain the final wall thickness for each vessel [22]. In addition, to evaluate the mechanisms underlying vascular remodeling, the immunofluorescence signal for α-SMA and CD31 was quantified in 5-micrometer-thick sections obtained from whole mouse lung. For each section, 5–8 different fields of lung parenchyma (area/frame 77,327.375 µm^2^) containing pulmonary small vessels with a diameter less than 50 μm were selected. Images were captured with a digital camera (QICAM) connected to a fluorescent microscope (Olympus BX60, Tokyo, Japan) at a final magnification of 200×. The expression levels of α-SMA and CD31 were measured by quantitative immunofluorescence. The fractional area occupied by the fluorescent signal and its intensity, expressed as IOD per area unit, was then evaluated using the software Image Pro Plus 7.0 (Media Cybernetics, Silver Spring, MD, USA) [23]. All images were acquired with pre-calibrated gain and exposure time. Non-specific fluorescence analysis was carried out by merging the emission signals from different excitation lengths on the same microscopic field. Measurements were performed by an operator blind to the experimental groups.

#### 2.8.2. Evaluation of Right Ventricular (RV) Hypertrophy

The RV hypertrophy, Fulton’s index, RV thickness and cross-sectional area (CSA) of cardiomyocytes were evaluated in a dedicated group of animals (saline *n* = 5 and HDM/CFA *n* = 5). At the end of the experimental protocol, the heart was carefully explanted and immediately placed in Krebs solution to stop the heart in diastolic phase. Subsequently, the right ventricle (RV) was separated from the left ventricle (LV) and interventricular septum (S); each portion of the heart was weighed separately. Fulton’s index was calculated using the formula RV/(LV + S). Additionally, RV samples were sliced into cross-sections, fixed in formalin, and embedded in paraffin. Three consecutive sections, 5 μm thick, were collected and stained with H&E staining, and RV hypertrophy was evaluated using the Nanozoomer Digital Pathology NDP.view2 Viewing software (Hamamatsu Photonics K.K, Hamamatsu City, Japan) for image analysis. To assess cell hypertrophy, for each sample, we measured 8 cardiomyocytes with a definite and clearly visible shape and each cross-sectional area (CM CSA) was morphometrically determined. The diameter was measured in transversally oriented myocytes localized in the endocardium and epicardium [24].

#### 2.8.3. Detection of α-SMA and Hypoxia-Inducible Factor 1-Alpha (Hif1-α) in Lung Parenchyma and Epithelium

α-SMA and hypoxia-inducible factor 1-alpha (Hif1-α) were detected by immunofluorescent staining (IF) performed on 5 µm thick paraffin-embedded sections of HDM/CFA or saline groups. A blocking solution composed of 5% donkey serum (D9663, Sigma Aldrich, St. Louis, MO, USA) and 5% goat serum (G9023, Sigma Aldrich, St. Louis, MO, USA) in PBS 1X (ThermoFisher Scientific, Waltham, MA, USA) was applied before primary antibody incubation. Mouse anti-α-SMA (Ab7817, 1:200, Abcam, Cambridge, UK) and rabbit anti-Hif1-α (Ab179483, 1:200, Abcam, Cambridge, UK) primary antibodies were incubated overnight at 4 °C. Goat anti-mouse Alexa Fluor555 (A-32727, 1:1000, ThermoFisher Scientific, Waltham, MA, USA) and donkey anti-rabbit Alexa Fluor488 (A-21206, 1:1000 ThermoFisher Scientific, Waltham, MA, USA) secondary antibodies were incubated at room temperature for 1 h. Nuclei were stained with DAPI (P36931, ThermoFisher Scientific, Waltham, MA, USA). IF images were acquired with the Zeiss LSM 710 confocal microscope (Carl Zeiss, Oberkochen, Germany).

### 2.9. Echocardiography

One day prior to the sacrifice, echocardiography was performed using the Vevo^®^ 3100 high-resolution Imaging System version 5.6.0 (FUJIFILM VisualSonics, Toronto, ON, Canada). Mice (saline *n* = 10 and HDM/CFA *n* = 15) were anesthetized with 4% isoflurane and dorsally positioned in a heat-controlled animal bed to maintain body temperature at 37 °C. Mice chests were shaved and ultrasound transmission gel (Cogel^®^ Ultrasound, Kaltek, Padua, Italy) was applied. Isoflurane concentration was reduced to a minimum (1–2%) to ensure constant and comparable heart rates during examination. Throughout the entire procedure, pressure, ventilation and temperature were continuously monitored. The thickness of the right ventricle (RVWT) was measured in parasternal long-axis view using 2D M-mode echocardiography [25]. Image analyses were conducted by two independent observers using the VevoLAB software package, Version 5.6.0 (FUJIFILM VisualSonics, Toronto, ON, Canada). The larger group size (*n* = 15) used for the HDM/CFA group was selected for echocardiographic assessment to reach a statistical power of 0.8, allowing for proper statistical comparison. The same mice group was used for the histological analysis. For all other analyses, smaller group sizes were used to limit the total number of animals while still enabling robust comparisons. This strategy reflects adherence to the 3Rs principle, particularly Reduction and Refinement, by using the minimum number of animals necessary to obtain reliable and reproducible results.

### 2.10. Statistical Analysis

Statistical analysis was performed using GraphPad Prism10 software version 10.6.1 for Windows (GraphPad Software, San Diego, CA, USA). Unpaired Student’s *t*-test for parametric analysis was used as detailed. All data are reported as the mean ± SD. Each animal was considered single experimental unit. A *p*-value ≤ 0.05 was considered statistically significant. * *p* < 0.05, ** *p* < 0.01, *** *p* < 0.001, and **** *p* < 0.0001. We checked for outliers using the ROUT method (Q = 1%) implemented in GraphPad Prism (GraphPad Software, San Diego, CA, USA). No animal was excluded.

## 3. Results

### 3.1. Repeated HDM Challenge in HDM/CFA-Sensitized Mice Promoted Mixed Inflammatory Infiltrate in BALF

HDM/CFA exposure (Figure 1A) produced a statistically significant elevation in the cellularity of BALF (Figure 1B–F). The number of WBCs was significantly higher in the HDM/CFA group compared to the saline group (Figure 1B, ** *p* <0.01), indicating a strong reaction of the immune system to stimuli. Furthermore, a significant increase in neutrophil levels was measured in the HDM/CFA group compared to the saline group (Figure 1C, ** *p* < 0.01), as well as an increase in eosinophils (Figure 1D, ** *p* < 0.01), indicating a mixed inflammatory phenotype. Moreover, both monocytes and lymphocytes showed significant increases in the HDM/CFA group compared to controls (Figure 1E,F, ** *p* < 0.01). The cytokine profile analyzed by using the Luminex multiplex immunoassay highlighted the elevation of interleukins such as IL-5, IL-4, and CCL11 (eotaxin), confirming an eosinophilic response, which is typically associated with Type 2 inflammation. In parallel, we observed a significant increase in IL-12p40, a subunit shared by the cytokines IL-12 and IL-23, both of which play critical roles in immune regulation. IL-12 is primarily involved in driving Th1 responses, which can indirectly promote neutrophilic activity [26]. IL-23 is closely associated with the expansion and stabilization of Th17 cells, which are key players in neutrophil-mediated immune responses [27]. The increased levels of IL-17, IL-9, IL-3 and others point to additional non-eosinophilic pathways, potentially involving neutrophilic inflammation and Th17-related immune mechanisms. This inflammatory profile is further compounded by the upregulation of IL-1beta, CCL4 (MIP-1beta), CCL3 (MIP-1alpha), and CCL5 (Rantes), cytokines known for their roles in recruiting and activating immune cells, such as macrophages and lymphocytes, and mediating inflammatory responses. This combination of eosinophilic, neutrophilic, lymphocytic, and macrophage activity confirms a complex, overlapping inflammatory profile, highlighting the interplay of innate and adaptive immunity observed in COPD patients (Supplementary Figures S1 and S2) [28].

### 3.2. Lung Remodeling and Pulmonary Function Impairment Are Promoted in HDM/CFA Mice

**Figure 2:**
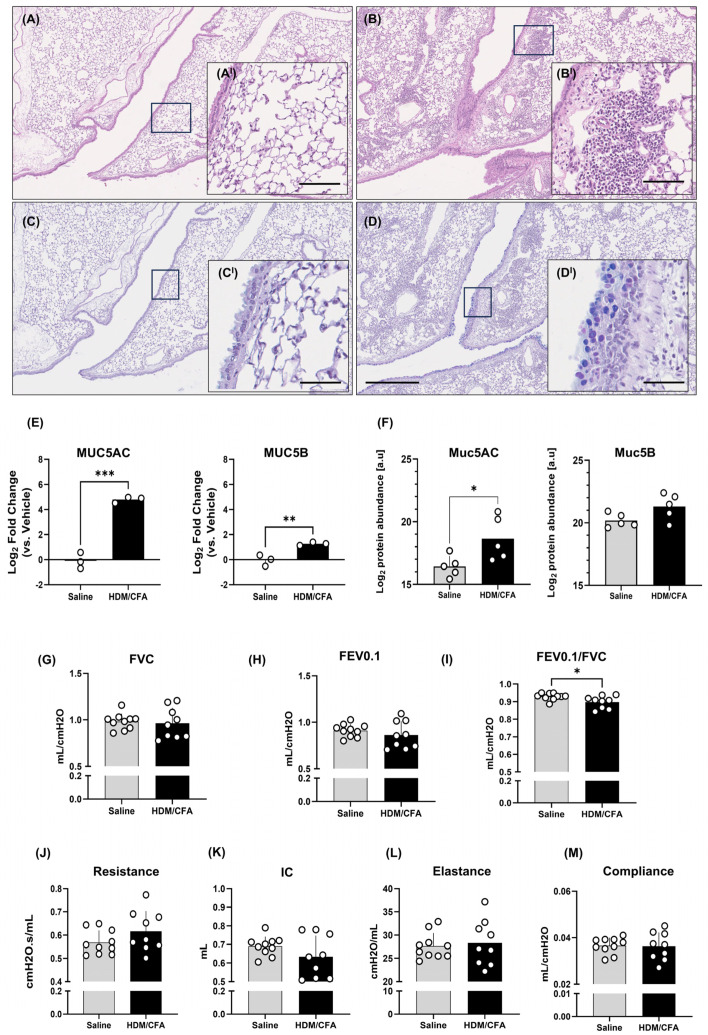
Assessment of mucus hypersecretion and pulmonary function following chronic HDM/CFA exposure. (**A**,**B**,**A^I^**,**B^I^**) Representative images of lung sections stained with H&E and (**C**,**D**,**C^I^**,**D^I^**) Alcian PAS. Scale bar = 500 µm in (**A**–**D**); scale bar = 100 µm in (**A^I^**,**B^I^**); scale bar = 50 µm in (**C^I^**,**D^I^**). Upregulation of (**E**) gene levels of *MUC5AC* and *MUC5B* and (**F**) proteomic levels of Muc5AC and Muc5B compared to the saline control group. For qPCR, the mean ± SD of Log2 fold change is shown with respect to the saline group. For proteomic analysis, the mean ± SD of Log2 protein abundance is shown with respect to the saline group. (**G**–**M**) Evaluation of lung function parameters in HDM/CFA and saline groups: FVC = forced vital capacity, Fev0.1 = forced expiratory volume in 0.1 s, Fev0.1/FVC = Fev0.1/FVC ratio, and IC = inspiratory capacity, resistance, compliance, and elastance. A reduction in pulmonary ventilation was seen in mice stimulated with HDM/CFA. Data are shown as mean ± SD. Statistical analysis was conducted using unpaired Student’s *t*-test in comparison with the saline group. * *p* < 0.05, ** *p* < 0.01, and *** *p* < 0.001.

Excessive mucus production and impaired pulmonary function are two key factors increasing the risk of mortality in COPD patients [29]. In the HDM/CFA group, foci of dense mononuclear infiltrates were detected, representing vascular and peri-bronchial inflammation; no inflammatory infiltrate was observed in the saline group (Figure 2A,B,A^I^,B^I)^). In stimulated animals, bronchial mucosal hyperplasia and goblet cell hypertrophy was observed in Alcian blue/PAS-stained sections, as well as bronchial muscular hypertrophy indicating significant structural changes in the bronchial walls compared to the saline group (Figure 2C,D,C^I^,D^I^). The observed bronchial alterations reflect ongoing adaptive responses to persistent inflammatory stimuli, leading to thickening and remodeling of the airway walls commonly seen in COPD patients [30]. Gene expression analysis and protein quantification of MUC5AC and MUC5B, the two main secreted mucins involved in mucus hyperproduction in COPD, were performed. The data indicate that gene expression of both *MUC5AC* and *MUC5B* was significantly upregulated in the HDM/CFA group compared to the saline group (Figure 2E, *** *p* < 0.001, ** *p* < 0.01). Moreover, *MUC5AC* showed a more substantial increase in fold change compared to MUC5B. In agreement with these data, proteomic analysis showed a significant increase in the protein level of Muc5AC and a trend of increase in MUC5B in mice stimulated with HDM/CFA with respect to the saline group (Figure 2F, * *p <* 0.05). The data obtained align with findings reported in human studies. Indeed, mass spectrometry quantification of Muc5AC and Muc5B in the sputum of the SPIROMICS population revealed a six-fold and two-fold increase in Muc5AC and Muc5B mean concentration, respectively, compared to the levels observed in healthy never-smokers [31]. The FlexiVent system was used to assess lung function in the HDM/CFA mouse model. The data show a reduction in FEV0.1, FVC and the surrogate of the human FEV1/FVC ratio, referred to as the FEV0.1/FVC ratio, in the HDM/CFA-stimulated group compared to the saline group (Figure 2G–I, * *p* < 0.05). These findings suggest that HDM challenge induced an obstructive pattern in HDM/CFA-sensitized mice [32]. Moreover, a trend of increase in airway resistance and a decrease in inspiratory capacity (IC) in the HDM/CFA group with respect to the saline group was observed (Figure 2J,K). The data indicate an alteration in lung function, evidenced by changes in inspired air volume and increased airway constriction, both of which are directly attributable to the stimulation protocol applied in this experimental model. The area between the inflation and deflation limbs of the pressure–volume loop was similar among all groups. No significant differences were present in the shape of the deflation limb of the pressure–volume loop as quantified by the shape parameter (K) (Supplementary Figure S3). Histological examination of HDM/CFA-treated mice revealed the absence of established emphysematous degeneration, a finding further supported by the absence of alteration in elastance and compliance (Figure 2L,M).

### 3.3. Vascular Remodeling in Lung Parenchyma and Evidence of Cardiac Tissue Alterations

**Figure 3:**
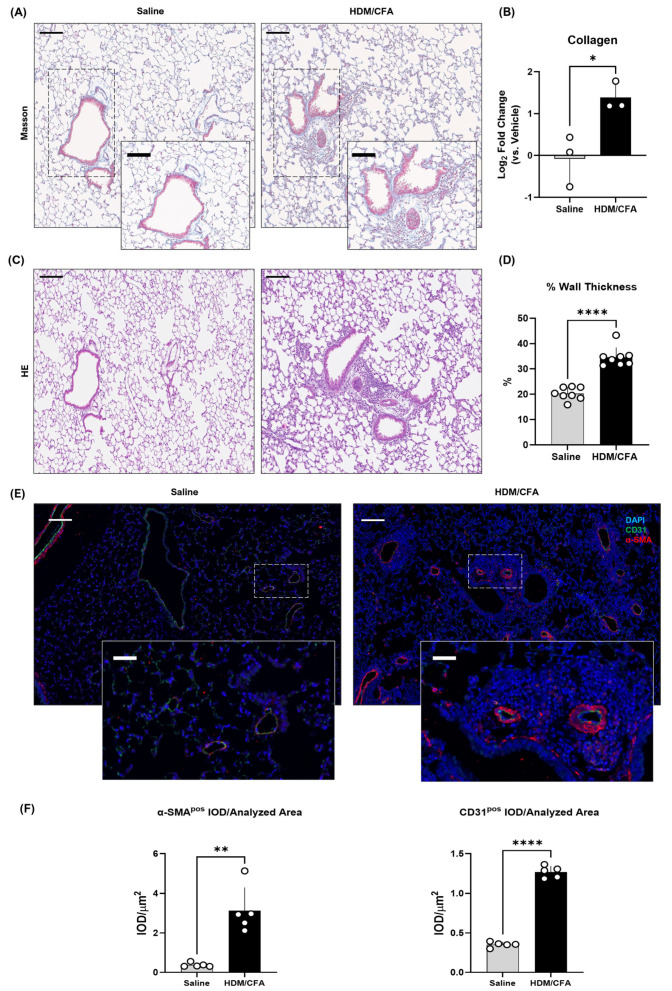
HDM/CFA chronic exposure induces vascular alteration and wall thickness in small pulmonary vessels. (**A**) Representative images of parenchymal lung sections of mice receiving HDM/CFA or saline solution stained with Masson’s and magnification of pulmonary vessels; scale bar 200 µm; scale bar magnification 100 µm. (**B**) mRNA expression analysis of collagen in lung homogenate of the HDM/CFA group relative to controls. The mean ± SD of Log_2_ FC is shown with respect to the saline group. (**C**) H&E staining was performed on lung sections from mice exposed to HDM/CFA or saline for 8 weeks to measure the wall thickness of small pulmonary vessels; scale bar 200 µm; scale bar magnification 100 µm. (**D**) The wall thickness percentage of pulmonary arteries (20–150 µm) was significantly increased in HDM-exposed mice in comparison with the saline group. Data are shown as the mean ± SD (**E**) Representative images of immunofluorescent staining performed on lung slices. Positive arterioles for CD31 are shown in green (FITC) and for α-SMA in red (TRITC). Nuclei, counterstained with DAPI, are visible in blue. Scale bar: 100 μm; scale bar magnification: 50 μm. (**F**) α-SMA and CD31 Integrated Optical Density (IOD) quantification on selected vessels fields in lung parenchyma of HDM/CFA revealed a significant increase in comparison with control mice. Data are shown as the mean ± SD. Statistical analysis was carried out using unpaired Student’s *t*-test in comparison with the saline group (* *p* < 0.05, ** *p* < 0.01; **** *p* < 0.0001).

**Figure 4:**
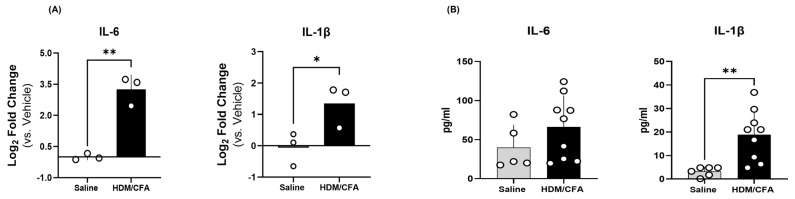
HDM/CFA exposure alters interleukin expression associated with vascular remodeling. (**A**) Upregulation of the expression of *IL-1β* and *IL-6* genes in HDM/CFA groups relative to controls. The mean ± SEM of Log2 FC is shown with respect to the saline group. (**B**) Quantification of protein levels of both IL-1β and IL-6 in the lung lysate of all experimental groups. Cytokine quantification is expressed in pg/mL. Data are shown as the mean ± SD. Statistical analysis was carried out using unpaired Student’s *t*-test vs. the saline control group (* *p* < 0.05, ** *p* < 0.01).

**Figure 5:**
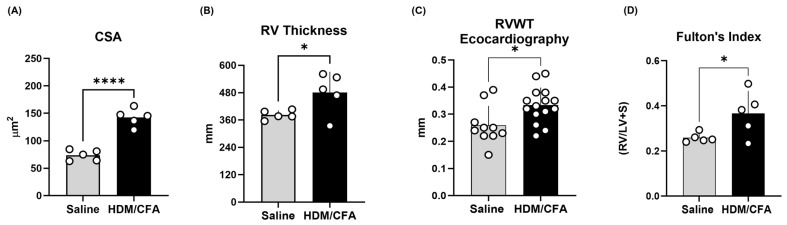
Signs of cardiac tissue alteration after 8-week HDM/CFA treatment. (**A**) The cross-sectional area (CSA) of cardiomyocytes shows a significant increase in stimulated mice vs. saline-treated mice. (**B**) Right ventricular (RV) wall thickness was increased in HDM/CFA-stimulated mice relative to the saline group. (**C**) Echocardiographic assessment of right ventricular wall thickness (RVWT) reveals an upward trend in HDM/CFA-stimulated mice compared to saline mice. (**D**) Fulton’s index, calculated as (RV/LV + S), indicates an increase in HDM/CFA-stimulated mice in comparison with the saline control group. Data are shown as the mean ± SD. Statistical analysis was performed using unpaired Student’s *t*-test vs. the saline control group (* *p* < 0.05, **** *p* < 0.0001).

Evidence of vascular remodeling was observed in HDM/CFA-sensitized mice. Analysis of H&E-stained sections highlighted a profound reorganization of vessel structure characterized by intima and media thickening and collagen deposition, as observed in Masson’s Trichrome-stained slices. The degree of remodeling in HDM/CFA mice varied among individuals and within vessels of the same mouse, from narrowing of the lumen to occluded vessels, while these features were absent in the control group (Figure 3A). Gene expression analysis confirmed the upregulation of a collagen gene (*Col3a1*) in the HDM/CFA group with respect to the saline group (Figure 3B, * *p* < 0.05). Quantitative analysis of vascular remodeling was performed according to the wall thickness percentage in H&E-stained sections (Supplementary Figure S4). Morphometric analysis displayed a significant increase in wall thickness of the HDM/CFA-treated group compared to the control group (Figure 3D, **** *p* < 0.0001). Additionally, immunofluorescence staining for α-SMA and CD31 was performed in subsequent sections (Figure 3E). A significant increase in the α-SMA and CD31 IOD was observed in the HDM/CFA group compared to the saline group (Figure 3F, ** *p* < 0.01, **** *p* < 0.0001, respectively). Furthermore, histological findings were confirmed by the expression analysis of markers involved in vascular remodeling such as IL-1β and IL-6. The gene expression levels of *IL-1β* and *IL-6* genes were significantly upregulated in the HDM/CFA group compared to controls (Figure 4A, ** *p* < 0.01, * *p* < 0.05). This was further validated by protein quantification using the Luminex technique, which detected a significant increase in the protein levels of IL-1β and IL-6 in the lung homogenate of the HDM/CFA group relative to controls (Figure 4B, ** *p* < 0.01). To analyze whether vascular remodeling affects cardiac tissues, we conducted H&E staining to assess the cross-sectional area (CSA) of cardiac myocytes in mice. The data showed significant cardiomyocyte hypertrophy in mice treated with HDM/CFA compared to the saline group (Figure 5A, **** *p* < 0.0001). Additionally, in the same animals, we observed an increase in the thickness of the right ventricle compared to the control group (Figure 5B, * *p* < 0.05). This finding was confirmed by echocardiography, which indicated an increased right ventricular thickness in HDM/CFA-treated mice with respect to the control group (Figure 5C, * *p* < 0.05). Ventricular hypertrophy was further confirmed by Fulton’s index analysis, which showed a significant increase in the treated group compared to the saline group (Figure 5D, * *p* < 0.05).

### 3.4. Activation of Key Proteins and Molecular Pathways in Response to Chronic HDM/CFA Exposure

**Figure 6:**
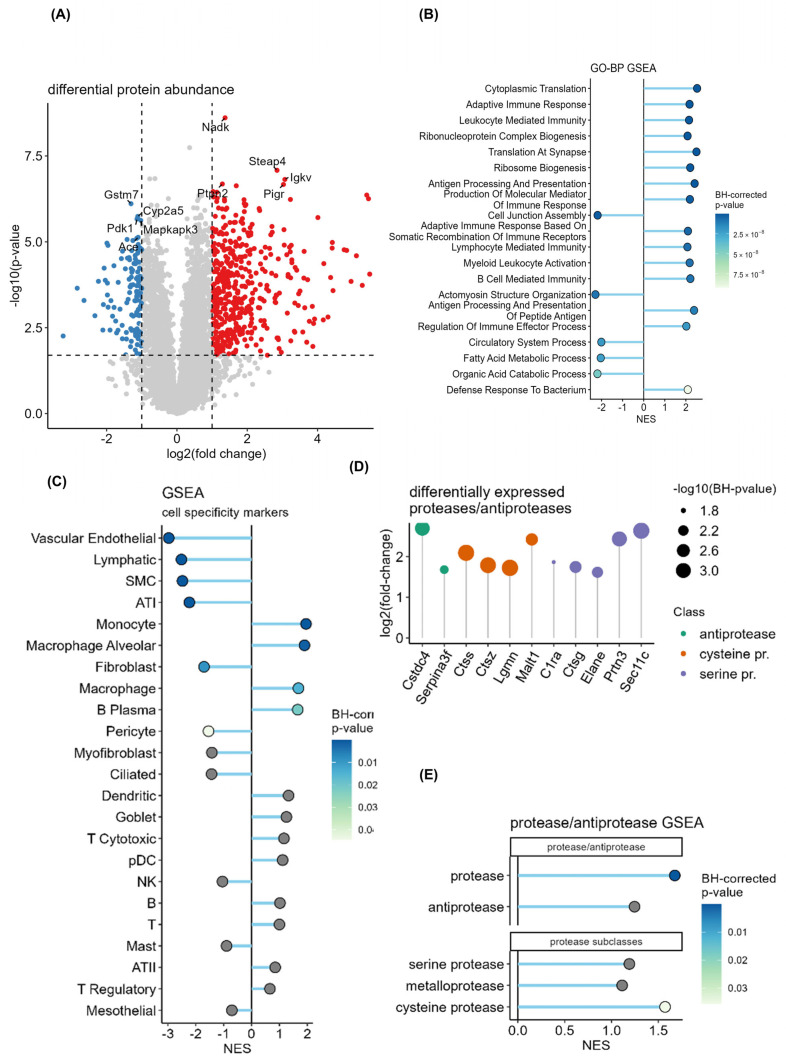
Proteomic analysis of HDM/CFA-treated and control mice. (**A**) Differentially expressed proteins between the lungs of HDM/CFA-treated and control mice. Each point represents a protein. The horizontal line indicates the significance threshold (BH-corrected *p*-value = 0.05), while the vertical lines indicate the foldchange thresholds (log2 fold change = ±1.5). Proteins significantly upregulated in HDM/CFA-treated lungs are shown in red, while significantly downregulated proteins are shown in blue. (**B**) The results of GO-BP gene set enrichment analysis. The top 20 most significantly enriched GO terms are shown, with the normalized enrichment score (NES) plotted on the *x*-axis. The color of the points corresponds to the statistical significance of the enrichment, with darker blue indicating smaller BH-corrected *p*-values. Positive NES values indicate enrichment of upregulated proteins, while negative NES values indicate enrichment of downregulated proteins. (**C**) The results of lung cell specificity gene set enrichment analysis. For each gene set, the NES value (*x*-axis line) and the adjusted *p*-value (dot color) are shown. Gray dots indicate non-significant enrichments (BH-corrected *p*-value > 0.05). (**D**) Differentially abundant proteases and antiproteases. Proteases with a BH-corrected *p*-value < 0.05 and log2FC > |0.5| are shown. (**E**) The results of GSEA analysis for protease and antiprotease gene sets. For each gene set, the NES value (*x*-axis line) as well as the adjusted *p*-value (dot color) are shown. Gray dots indicate non-significant results (BH-corrected *p*-value > 0.05).

Differential protein abundance analysis between HDM/CFA- and saline-treated samples, identified 481 upregulated (log2 fold change > 1 and BH-corrected *p*-value < 0.05) and 132 downregulated proteins (log2 fold change < −1 and BH-corrected *p*-value < 0.05) (Supplementary Table S1). Among the most upregulated proteins, we identified the metallo reductase Steap4, a protein known to increase its expression in response to inflammatory cytokines [33]. IGKV (immunoglobulin kappa variable) upregulation has been observed in COPD lung proteomics together with other immunoglobulin (Ig)-related genes [34]. PIGR, which is involved in specific immune defense and inflammation, has been previously reported to be upregulated in the sputum and bronchial and alveolar epithelium of smokers and further increased in the alveolar area in mild to moderate COPD [35]. In contrast, severe COPD is associated with a significant decrease in PIGR expression in the bronchial epithelium. This reduction correlated with a decrease in lung function (FEV1) and a more severe disease state [36]. GSTM7 (glutathione S-transferase, mu 7) plays a role in cellular detoxification and emerged as one of the most downregulated proteins. Glutathione transferases (GSTs) are involved in COPD through their role in detoxifying harmful compounds from cigarette smoke, with genetic variations affecting an individual’s susceptibility to the disease [37]. Gene set enrichment analysis using GO-BP showed a positive enrichment of several immune-related terms, including adaptive immune response and leucocyte-mediated immunity. In contrast, the downregulated proteins were enriched in terms such as cell junction assembly, actomyosin structure organization and circulatory systems process (Figure 6B; Supplementary Table S2). To further explore how the dysregulated proteins relate to lung cell types, we performed GSEA using custom gene sets of lung cell markers derived from a COPD scRNA-seq study [20]. This analysis identified the gene set of vascular endothelial cell markers as the most significantly downregulated (NES: −2.97; BH-corrected *p*-value: 2.7 × 10^−14^), followed by the gene set of lymphatic cells (Figure 6C). Among the leading proteins driving endothelial cell enrichment, we identified the arterial marker molecule ephrin B2 (Efnb2—log2 fold change: −1.15; BH-corrected *p*-value: 0.0023). Instead, among the lymphatic markers, we observed the presence of Lymphatic Vessel Endothelial Hyaluronan Receptor 1 (Lyve1—log2 fold change: −1; BH-corrected *p*-value: 0.003). In agreement with the GO-BP enrichment analysis, markers for monocytes/macrophages and B cells were positively enriched, further supporting immune system activation. Among the dysregulated cell junction-related proteins identified from the GO-BP analysis, we observed significant downregulation of Fermitin family homolog 2 (Fermt2—log2 fold change: −1.88; BH-corrected *p*-value: 0.0034), which is a kindlin protein reported to be involved in vascular stabilization [38,39]. Of note, other proteins that contribute to both cell junction architecture and endothelial function were downregulated, albeit to a lesser extent. For example, Cadherin-5 (Cdh5—log2 fold change: −0.89; BH-corrected *p*-value: 0.0004), which encodes the vascular endothelial (VE), a cadherin protein specifically expressed in endothelial cells and Claudin-5 (Cldn5—log2 fold change: −0.82; BH-corrected *p*-value: 0.0014), is a key component of tight junctions that maintains the integrity and selective permeability of the endothelial barrier. Moreover, Integrin beta-1, which plays a critical role in stabilizing the VE cadherin at adherens junctions and maintaining junctional integrity, also showed reduced expression (Itgb1—log2 fold change: −0.63; BH-corrected *p*-value: 0.0013). Given the reported imbalance between protease and antiprotease in COPD pathogenesis, particularly in emphysema [40], we sought to investigate the dysregulation of the proteins belonging to these functional classes in our model. To this end, we retrieved proteins classified as proteases and antiproteases, as well as individual protease subclasses, from the PANTHER database [41] and analyzed their dysregulation in our proteomic dataset (Figure 6D). Among the top differentially abundant proteins, we identified five serine proteases, including Neutrophil Elastase (Elane), Cathepsin G (Ctsg), and Proteinase 3 (Prtn3), all known to be involved in the destruction of the alveolar tissue in COPD [40]. Among the cysteine proteases, we identified Cathepsin S (Ctss), a lysosomal protein previously reported as a contributor to the pathogenesis and progression of a variety of pulmonary diseases, including COPD [42], and shown to correlate positively with smoking [43]. To further investigate the role of the protease/antiprotease system in our murine model of lung inflammation, we performed gene set enrichment analysis and identified significant positive enrichment of proteases (NES: 1.68; BH-corrected *p*-value 0.00004). When analyzing the protease subclasses separately, we observed significant positive enrichment of the cysteine protease gene set (NES: 1.57; BH-corrected *p*-value 0.04) (Figure 6E). Taken together, these findings indicate that HDM/CFA exposure induces profound changes in lung tissue, characterized by robust immune activation, alongside endothelial dysfunction and dysregulation of protease-related pathways; however, overt alveolar destruction or emphysema is not evident in the current histological analysis.

### 3.5. Role of Hypoxia in the Development and Progression of Airway and Vascular Remodeling

**Figure 7:**
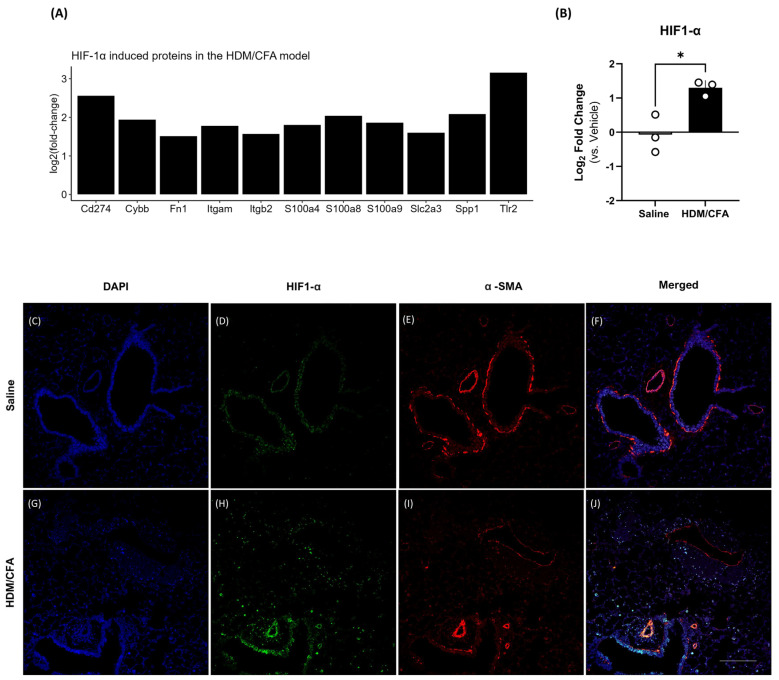
HIF1-α protein and mRNA expression in mouse lung sections. (**A**) Quantification of Hif1-α-induced proteins in the HDM/CFA model. (**B**) Expression levels of the *Hif1-α* gene. The mean ± SEM of Log2 FC is shown with respect to the saline group. Statistical analysis was carried out using unpaired Student’s *t*-test vs. the saline control group (* *p* < 0.05). (**C**–**J**) Representative images of immunofluorescent staining performed on lung slices. Hif1-α-positive lung parenchyma and bronchial epithelium are stained in green and positive arterioles for the α-SMA antibody in red. Nuclei, counterstained with DAPI, are visible in blue. Scale bar: 200 μm.

The hypoxic pathway is closely related to the chronic inflammatory response and HIF1-α plays a central role both in the physiological response to hypoxia and in various pathological processes associated with COPD and PH [44]. To investigate HIF1-α-mediated dysregulation of the proteomic profile in the HDM/CFA model, we retrieved a list of *HIF1-α*-induced genes from CollectTRI, a collection of signed *TF–*gene interactions [45], and performed regulon enrichment analysis. This analysis revealed significant positive enrichment (NES: 1.32; *p*-value = 0.04). The most significant upregulated proteins contributing to the enrichment score (log2 fold change > 1.5 and BH-corrected *p*-value < 0.05) are reported in Figure 7A. Among these, toll like receptor 2 (Tlr2) was the most upregulated protein (log2 fold change = 3.2; BH-corrected *p*-value = 0.001), followed by Programmed Death-Ligand 1 (Cd274—log2 fold change = 2.6; BH-corrected *p*-value = 0.001), both inflammatory mediators (Figure 7A). To complement the analysis of *HIF1-α*-regulated genes, we also evaluated *HIF1-α* itself by combining gene expression profiling with protein-level measurements. Gene expression analysis showed significantly higher *HIF1-α* transcript levels in lung homogenate of the HDM/CFA group compared to the controls (Figure 7B, * *p* < 0.05). Additionally, the immunofluorescence signal for HIF1-α increased in lung sections of HDM/CFA mice compared to saline mice. It appeared to be localized mainly in bronchial epithelial cells, parenchyma and smooth muscle cells surrounding remodeled vessels, as confirmed by the colocalization with α-SMA (Figure 7C–J).

## 4. Discussion

COPD is recognized as a highly heterogenous condition, as shown by its varied clinical manifestation, and currently available therapies for its treatment remain poor. Accurate detection of subgroups is needed to develop novel personalized therapies and to guide choices on existing treatments. COPD phenotypes are usually classified based on the severity of airflow limitation; several methods have been proposed to develop more comprehensive approaches considering multiple clinical variables [46], the detection of COPD subtypes by image biomarkers [47], and the frequency of exacerbations [48]. However, the identified clusters are not consistent between different studies and populations and may not be stable over time; therefore, they have not proved to be useful in treatment selection [49]. Identification of endotypes based on the underlying pathophysiological mechanisms rather than clinical signs might guide the future of drug development to treat COPD [50]. Specific endotypes are the result of a complex interaction between genetic background and exposure to different risk factors. Mechanisms driving tobacco exposure toxicity have been extensively studied and the screening of novel potential pharmacological treatments for COPD relies on rodent models of smoke exposure. However, up to 25–45% of patients with COPD have never smoked and the burden of non-smoker COPD is increasing. The development of specific therapies targeting patient subpopulations requires further understanding of the pathophysiology of COPD endotypes and a parallel improvement in preclinical models recapitulating them. Exposure to grain dust on farms, fumes and dust in factories and a history of asthma can contribute to the development of COPD through different pathophysiological processes compared to cigarette smoke. In the present study, a murine model was employed to investigate the pulmonary inflammatory response that may be particularly relevant for a subset of patients within the heterogeneous COPD population. Mice sensitized with HDM in CFA were subjected to repeated HDM challenges over a six-week period, resulting in the induction of a complex inflammatory milieu within the lung tissue. Repeated exposure to the allergen elicited robust leukocyte recruitment, with neutrophils representing the predominant cell population in BALF. Correspondingly, elevated levels of interleukin-1 beta (IL-1β), a potent neutrophil chemoattractant, were detected. Proteomic analysis revealed that activated neutrophils released a spectrum of pro-inflammatory mediators, including cytokines, chemokines, and proteolytic enzymes such as neutrophil elastase, cathepsin G, and proteinase 3. These factors are implicated in extracellular matrix remodeling and further immune cell recruitment [51]. Gene set enrichment analysis (GSEA) revealed a significant enrichment of monocyte and alveolar macrophage markers in the pulmonary tissue of mice subjected to house dust mite (HDM) challenge. These immune cell populations are known to contribute to the secretion of matrix metalloproteinases (MMPs), which are key mediators of extracellular matrix degradation and play a pivotal role in tissue injury and structural remodeling of the lung parenchyma. As illustrated in Figure 6E, the model reproduces the protease–antiprotease imbalance, a hallmark of chronic obstructive pulmonary disease (COPD), although no overt signs of alveolar damage were detected. COPD pathophysiology typically evolves over extended timeframes not fully captured here. Protease-antiprotease imbalance is considered an early and key event in the development of COPD, as it is a fundamental mechanism leading to the progressive destruction of lung tissue. This imbalance can result from genetic factors, such as a deficiency in the antiprotease alpha-1-antitrypsin (AAT), or from an overproduction of proteases stimulated by smoking and other environmental factors. Dysregulated activity of metalloproteinases has been implicated in the pathological remodeling of alveolar structures, underscoring their contribution to disease progression [52]. In HDM-challenged mice, the increase in innate immune cell populations within BALF was accompanied by a significant elevation in lymphocyte counts and heightened levels of pro-inflammatory cytokines, including interleukin-17 (IL-17), interferon-γ-inducible protein 10 (IP-10), and interleukin-9 (IL-9). GSEA further confirmed positive enrichment of multiple immune-related Gene Ontology (GO) terms associated with the adaptive immune response. Moreover, transcriptional markers indicative of monocytes/macrophages, B cells and T cells were significantly enriched. These findings corroborate that the inflammatory profile observed in the model mirrors that of specific subsets within the broader spectrum of human COPD, where chronic airway inflammation is driven by the activation of both innate and adaptive immune pathways. This is typified by the infiltration of CD8^+^ cytotoxic T lymphocytes, B cells and CD4^+^ T helper cells of the Th1 and Th17 lineages into the lung parenchyma, contributing to persistent inflammation and tissue damage [53]. Indeed, the increased CD8^+^ cells in the peripheral airways of COPD patients have been related to smoking-induced airway limitation [54]. T cells contribute to lung tissue destruction in COPD both directly, through cytotoxic mechanisms, and indirectly, by activating macrophages [55] and amplifying the inflammatory cascade. This dual mode of action underscores a mechanistic link between the immune-mediated inflammatory response and the structural remodeling of the lung parenchyma. Interestingly, analysis of histological sections of HDM-challenged mice highlighted areas of dense mononuclear infiltrates around vessels adjacent to bronchi, bronchial mucosal hyperplasia, goblet cell hypertrophy, and bronchial muscular hypertrophy, indicating significant structural changes in the bronchial walls. Airway remodeling in the model was associated with a significant reduction in FEV0.1/FVC and elevated levels of the gel-forming mucins MUC5AC and MUC5B, reflecting key pathological features that are particularly prominent in COPD patients presenting with chronic bronchitis. These findings are consistent with data from the SPIROMICS cohort, which quantified sputum mucins across individuals with varying COPD severity. In healthy never-smokers, MUC5B was the predominant mucin, whereas in COPD patients, MUC5AC levels were markedly increased (approximately six-fold), resulting in a higher MUC5AC/MUC5B ratio. Elevated MUC5AC concentrations correlated with reduced lung function (lower FEV_1_/FVC), supporting a link between mucin dysregulation and disease severity [31]. Histological analysis revealed that structural remodeling in the HDM-challenged model extended beyond the bronchial compartment to include the pulmonary vasculature. Pulmonary arterioles exhibited marked wall thickening and luminal narrowing. Our findings indicate that α-SMA immunofluorescence staining revealed two key processes: further muscularization of vessels that were already muscularized and the emergence of muscularization in previously non-muscularized vessels. These changes suggest active remodeling of the vascular structure under the studied conditions. Although the precise molecular mechanisms underlying COPD and PH progression remain incompletely defined, both conditions share common risk factors and chronic inflammation, which over time promote airway and vascular remodeling. The hypoxic signaling pathway is closely intertwined with this inflammatory milieu, with hypoxia-inducible factor1-α (HIF1-α) acting as a central regulator. Under hypoxic conditions, HIF1-α stabilizes and translocate to the nucleus, activating the transcription of multiple genes involved in angiogenesis, vascular remodeling, and metabolic adaptation. HIF1-α is consistently elevated in COPD patients and correlates with airway and vascular changes, positioning it as a potential biomarker and therapeutic target for COPD progression and associated pulmonary hypertension (PH). Gene set enrichment analysis (GSEA) further indicated the downregulation of vascular endothelial markers, supporting the hypothesis that chronic inflammation may contribute to endothelial dysfunction. Emerging evidence in the literature highlights the critical role of the endothelium in COPD pathogenesis, with studies reporting increased endothelial apoptosis in patient lung tissue. Experimental evidence demonstrates that Sugen 5416, a vascular endothelial growth factor receptor antagonist, compromises pulmonary endothelial homeostasis, leading to the development of both pulmonary hypertension (PH) and emphysematous changes [56]. These findings underscore the central importance of endothelial integrity in maintaining alveolar structure and vascular function, suggesting that disruption of this barrier may represent a common mechanistic link between vascular remodeling and parenchymal destruction. Beyond pulmonary circulation, systemic endothelial dysfunction has been consistently reported in chronic obstructive pulmonary disease (COPD) [57], implicating a generalized vascular pathology that extends beyond the lungs. Clinically, this impairment may contribute to systemic arterial hypertension, left ventricular hypertrophy, and diastolic dysfunction, thereby explaining the high prevalence of cardiovascular comorbidities and adverse outcomes in COPD population. These observations highlight the need for integrated therapeutic strategies that target endothelial health, not only to mitigate pulmonary vascular disease but also to reduce cardiovascular risk and improve overall prognosis in COPD. The present study has several limitations that should be acknowledged. First, the HDM/CFA protocol does not represent a full experimental model of COPD, a disease that is highly heterogeneous, multifactorial, and typically develops over decades in humans. Key hallmarks of classical COPD, such as established emphysema, irreversible airflow obstruction, and a progressive decline in lung function, were not recapitulated within the timeframe of this model. Rather than attempting to comprehensively model COPD pathogenesis, the aim of this study was to reproduce specific inflammatory endotypes that are increasingly recognized within subsets of COPD patients, particularly those characterized by mixed eosinophilic–neutrophilic inflammation and prominent airway remodeling. Accordingly, this model was designed as a reductionist and mechanistic platform to dissect the impact of chronic inflammation per se on airway and pulmonary vascular remodeling, independently of cigarette smoke exposure and advanced parenchymal destruction. While this approach limits direct disease translation, it enables focused investigation of inflammation-driven structural and molecular changes that may contribute to pulmonary vascular remodeling and right ventricular alterations observed in COPD-associated pulmonary hypertension. Additional limitations include the use of a single mouse strain, a single sensitization and challenge protocol, and the absence of longitudinal assessment, which precludes evaluation of disease progression or reversibility. Furthermore, sex-specific effects were not addressed, as only female mice were used. Finally, extrapolation of these findings to the human condition should be made with caution, given interspecies differences and the simplified nature of the experimental system. Despite these limitations, the model provides a valuable experimental framework to explore inflammation-driven mechanisms relevant to selected COPD endotypes and to identify molecular pathways linking airway inflammation with vascular remodeling.

## Data Availability

All relevant data are provided in the main manuscript and the accompanying Supplementary Materials.

## Supplementary Materials

The following supporting information can be downloaded at https://www.mdpi.com/article/10.3390/biomedicines14061359/s1: Figure S1: Cytokines; Figure S2: Cytokines; Figure S3: Individual (A) and group average (B) PV loops; Figure S4: Morphometric measure of wall thickness of pulmonary arteries Table S1: Table of DEGs; Table S2: Table of GSEA_GO_BP.

## Abbreviations

The following abbreviations are used in this manuscript:

COPD	Chronic obstructive pulmonary disease
CVD	Cardiovascular disease
PH	Pulmonary hypertension
HDM	House dust mite Farinae
CFA	Freund’s Complete Adjuvant
IN	Intranasal injection
SC	Subcutaneous injection
BAL	Broncho-alveolar lavage
BALF	Broncho-alveolar lavage fluid
RV	Right ventricle
RVWT	Right ventricle wall thickness
IHC	Immunohistochemistry
IF	Immunofluorescence
IOD	Integrated Optical Density
H&E	Hematoxylin and Eosin
