# Supplementary figures and images for: From Airways to Arteries: Dissecting the Inflammatory Mechanisms of Pulmonary Vascular Remodeling in a Murine Model of Chronic Airway Inflammation
Source: Biomedicines. 2026 Jun 17;14(6):1359. doi: 10.3390/biomedicines14061359 (PMC13296976; doi:10.3390/biomedicines14061359)

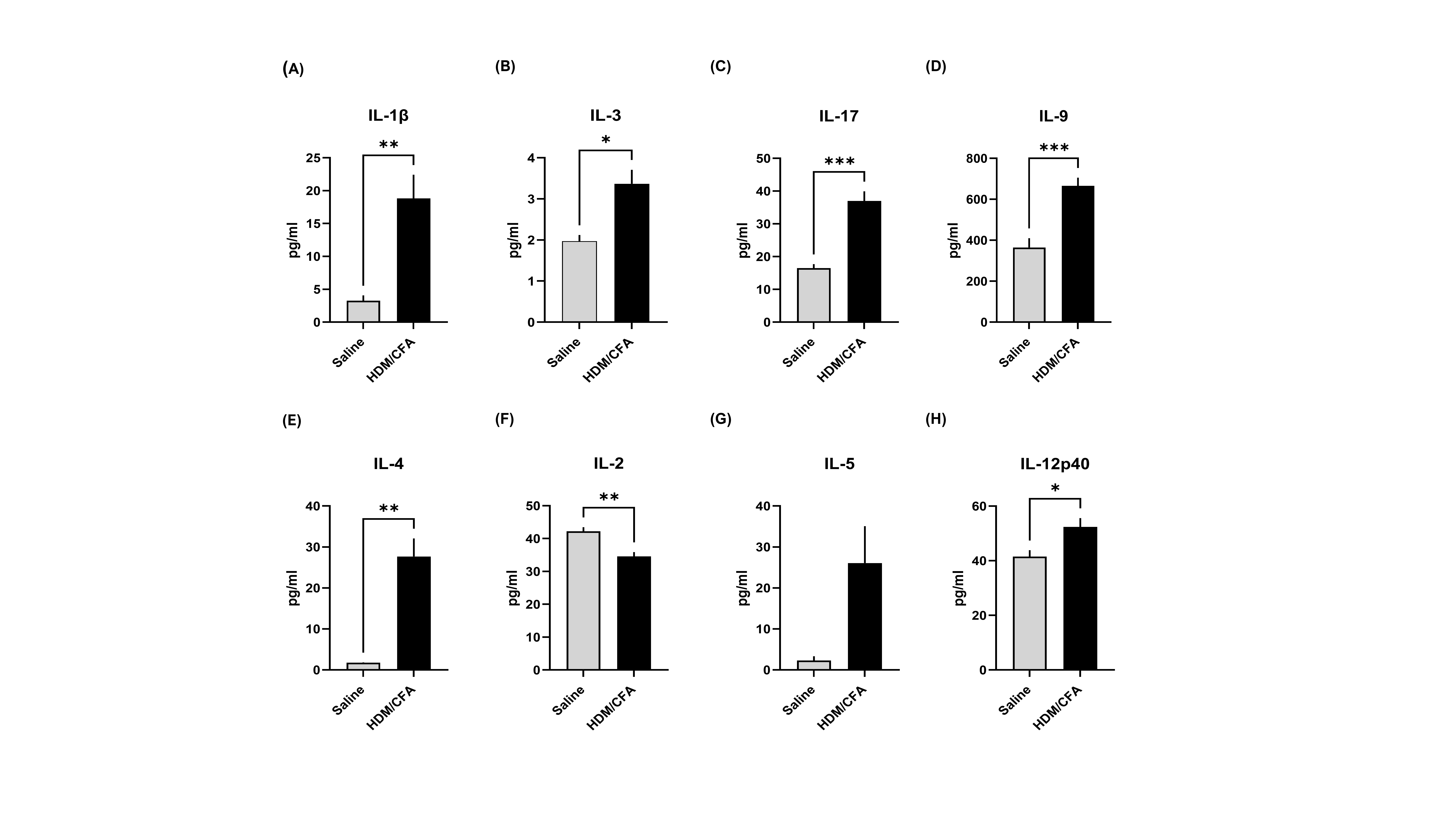

Supplement: Supplementary file 1 [file biomedicines-14-01359-s001.zip › Supplementary Figure S1.png]

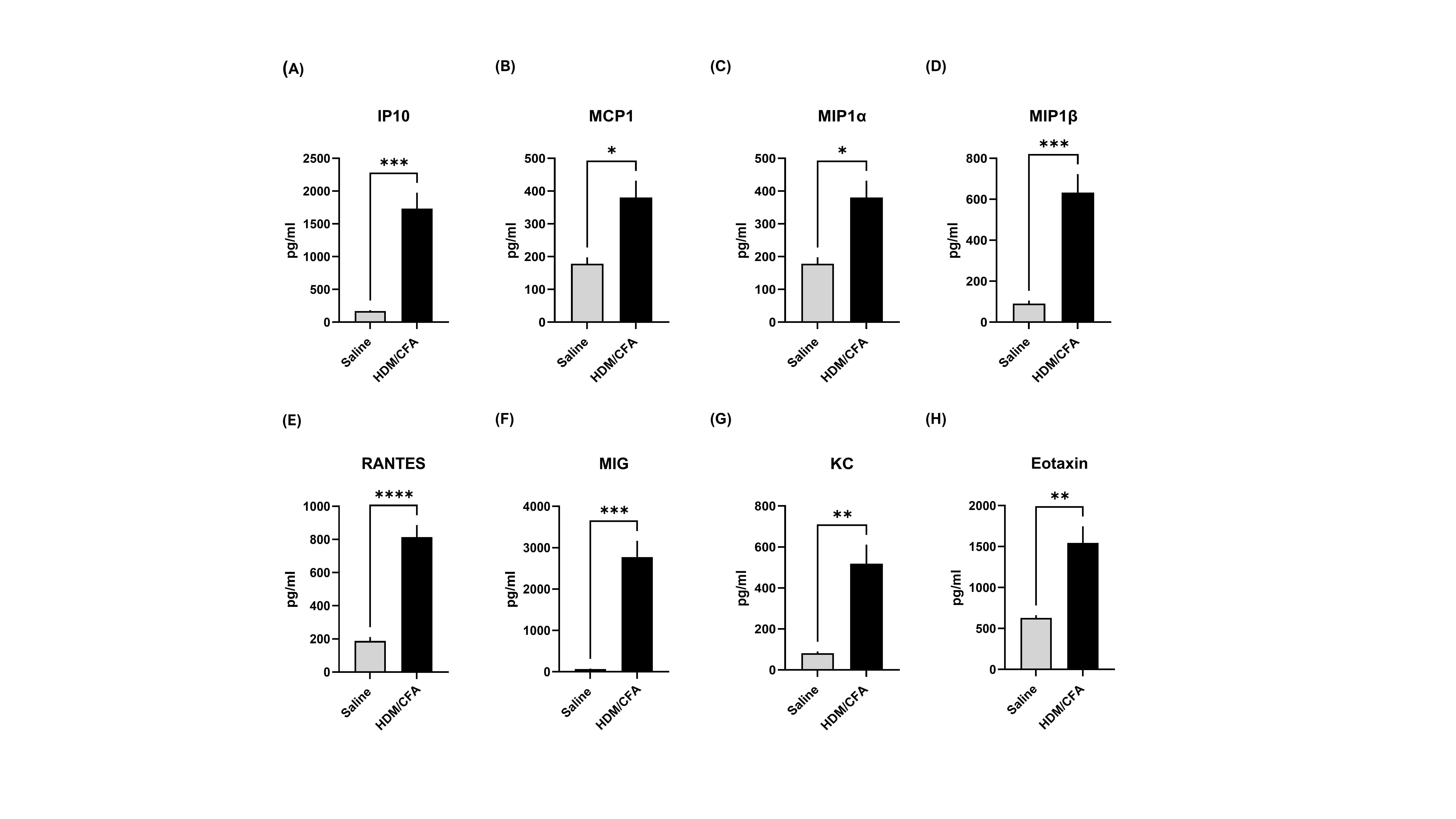

Supplement: Supplementary file 1 [file biomedicines-14-01359-s001.zip › Supplementary Figure S2.png]

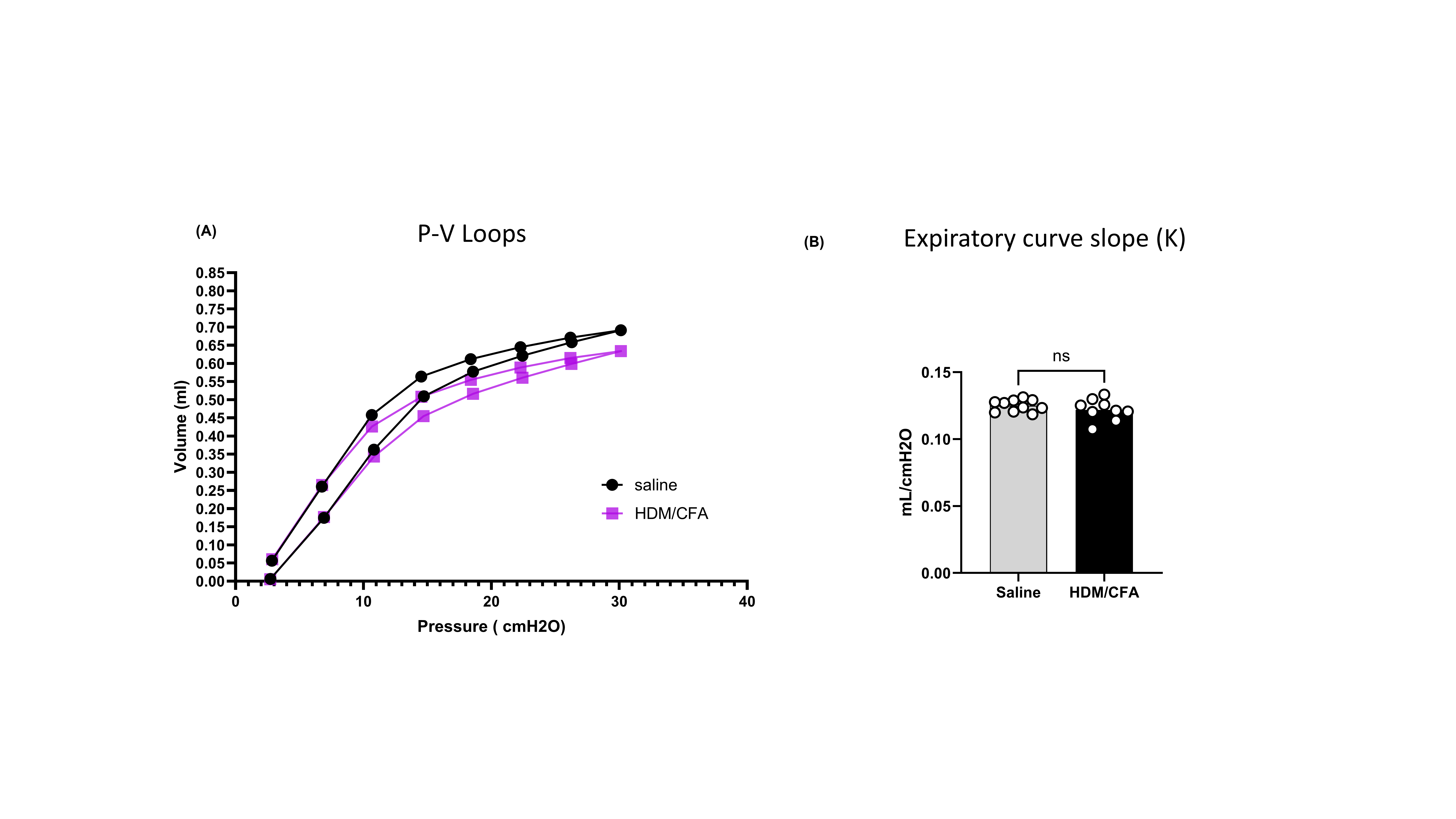

Supplement: Supplementary file 1 [file biomedicines-14-01359-s001.zip › Supplementary Figure S3.png]

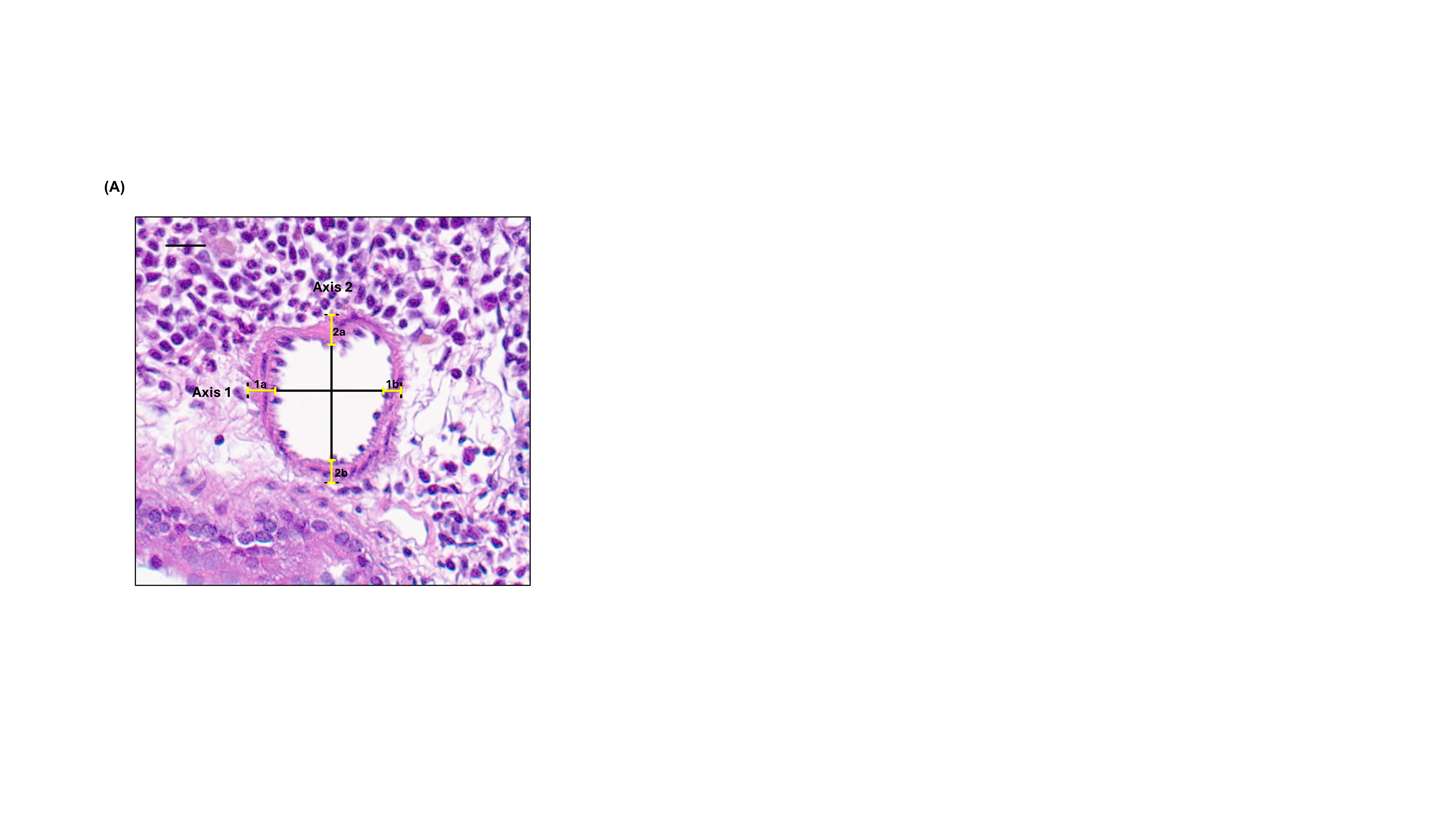

Supplement: Supplementary file 1 [file biomedicines-14-01359-s001.zip › Supplementary Figure S4.png]
